# Multitargeting Compounds: A Promising Strategy to Overcome Multi-Drug Resistant Tuberculosis

**DOI:** 10.3390/molecules25051239

**Published:** 2020-03-09

**Authors:** Giovanni Stelitano, José Camilla Sammartino, Laurent Roberto Chiarelli

**Affiliations:** 1 Department of Biology and Biotechnology “Lazzaro Spallanzani”, University of Pavia, 27100 Pavia, Italy; giovanni.stelitano01@universitadipavia.it (G.S.); jose.sammartino@iusspavia.it (J.C.S.); 2 IUSS-University School for Advanced Studies, 27100 Pavia, Italy

**Keywords:** tuberculosis, drug resistance, drug targets, polypharmacology, multitargeting compounds

## Abstract

Tuberculosis is still an urgent global health problem, mainly due to the spread of multi-drug resistant *M. tuberculosis* strains, which lead to the need of new more efficient drugs. A strategy to overcome the problem of the resistance insurgence could be the polypharmacology approach, to develop single molecules that act on different targets. Polypharmacology could have features that make it an approach more effective than the classical polypharmacy, in which different drugs with high affinity for one target are taken together. Firstly, for a compound that has multiple targets, the probability of development of resistance should be considerably reduced. Moreover, such compounds should have higher efficacy, and could show synergic effects. Lastly, the use of a single molecule should be conceivably associated with a lower risk of side effects, and problems of drug–drug interaction. Indeed, the multitargeting approach for the development of novel antitubercular drugs have gained great interest in recent years. This review article aims to provide an overview of the most recent and promising multitargeting antitubercular drug candidates.

## 1. Introduction

Tuberculosis (TB) is an airborne infectious disease caused by the bacillus *Mycobacterium tuberculosis* (MTB). To date, MTB still represents one of the most dangerous pathogens, claiming millions of lives each year worldwide. As reported by the WHO Global report of 2019, in 2018 at least 10 million people fell ill with TB, of which 1.2 million died among HIV-negative subjects, but the number grows with an additional 251,000 deaths among HIV-positive patients [1]. Even if the numbers are still high, there is an overall reduction in recent years, this can be correlated to the improvements in the EndTB Strategy, that started in 2015, whose aim is to reduce by 80% the incidence of TB worldwide and by 90% the number of deaths caused by this single infectious agent before 2030 [1].

Understanding the dynamics of MTB transmission is fundamental to control and prevent TB spreading, especially in high burden countries where this pathogen is still endemic. Genotyping and spoligotyping are established strategies for the molecular characterization and identification of MTB strains [2]. The complex interactions between MTB and the host during first contact, infection, and persistence are yet to be fully understood. The human body response to infectious agents calls for the mobilization of the innate immune system cells, but MTB can evade the immune response and persist in the human body thanks to its high genome plasticity [3]. In addition, upon MTB exposure, only a small percentage of patients will develop an active form of TB, while the majority of them will have a latent TB infection (LTBI) [4]. LTBI is a persistent immune response to MTB antigens in the absence of clinical symptoms of TB and can be diagnosed with the interferon-γ release assay (IGRA) and the tuberculin skin tests (TST), but the diagnosis of LTBI is not predictive of developing active TB [5]. Generally, LTBI can switch into the active form of TB in case of a strong immune system weakening, such as in case of HIV co-infection, or autoimmune diseases, but it can occur in not-high-risk patients, as well [5].

Treatment for active drug-susceptible TB is expensive and long, taking up to six months of daily doses of four first line drugs: Isoniazid, rifampicin, ethambutol, and pyrazinamide. Treatment success rate is generally higher when the patient follows the treatment to its completion. Low treatment success can depend on multiple factors, poor counseling during the treatment phase with consequent drop-out, high-risk populations (refugees, poor), co-morbidity with other pathologies [6]. For these reasons, supporting and informing the patients is pivotal. Furthermore, treatment outcomes depend on the spreading of MTB drug-resistant strains, for which the canonical therapy does not work [7]. Drug susceptibility tests should be performed, not only before, but also during therapy, to allow for an early identification of developing drug-resistant TB (DR-TB) [7].

Therefore, DR-TB should be carefully treated based on the resistance phenotype of the MTB drug-resistant strain. As recommended by the WHO consolidated guidelines on drug-resistant tuberculosis treatment of 2019, rifampicin-susceptible and isoniazid-resistant TB should be treated with daily doses of rifampicin, ethambutol, pyrazinamide, and levofloxacin or other fluoroquinolones [8]. 

Poor management of DR-TB can develop in multi-drug resistant TB (MDR-TB) with acquired rifampicin-resistance (RR-TB) with or without resistance to other first line drugs. MDR-TB/RR-TB treatment can be highly challenging, usually needing personalized strategies for each patient [9]. 

Treatment regimen for MDR-TB requires eight months of daily administration of pyrazinamide in association with at least four more second-line drugs. The duration of the treatment can vary from 12 to 20 months, but mostly depends on the patient response and TB evolution [9]. In the treatment of MDR-TB, fluoroquinolones, injectable anti-TB drugs, ethionamide, and cycloserine should be used in association to pyrazinamide for the first intensive phase, while pyrazinamide should be continued for the entirety of the treatment [9]. If cycloserine cannot be used, the *para*-aminosalicylic acid (PAS) should be used instead [9]. Streptomycin should be used as a second-line drug only for amikacin-resistant MDR-TB, as it correlates with a reduced treatment success rate [8]. 

The spreading of MDR MTB strains has become a major health problem worldwide, especially in high burden countries, where in depth analysis, exhaustive follow-up information retrieval, and organic procedures are challenging [10]. Furthermore, due to poor management of treatment administration and overall inadequate antibiotic distribution, MDR-TB can dramatically evolve into extensively drug resistant TB (XDR-TB) resistant to isoniazid and rifampicin, associated with resistance to at least one fluoroquinolone and one injectable second line drug [11]. The use of multiple drugs in combination over a long period of time, in association with uninformed antibiotic administration, scarce thorough analysis, inadequate patients support and follow-up, paves the way for the onset of drug-resistance. Currently, there is the need to find new and more efficient ways to fight MTB infections, that can help bypass the problems of the current therapy. The tempting idea behind the concept of multitargeting drugs is the reduction of the causalities behind the development of drug resistance, which could help also reduce the time of antibiotics exposure and the number of antibiotics administrated to the patients, further reducing the risk of developing mechanism of resistance and consequently enhancing the possibility of complete recovery.

## 2. How Polypharmacology Can Help in Fighting MDR-TB?

Drug resistance mechanisms are a main problem to face in fighting MDR- and XDR-TB strains. A pathogen, indeed, requires often just a single base mutation to became resilient to antibiotics or chemotherapeutics. The development of a single pharmaceutical molecule that acts on different targets is a possible strategy to bypass this problem. This multitargeting system is known as “polypharmacology”. SQ109, for example, is a promising multitarget drug that putatively hits four different targets in the respiratory chain of *M. tuberculosis* [12].

Polypharmacology is in contraposition with the more classical “polypharmacy”, the use of different drugs, each with high affinity for one target, that are taken together as cocktails or multicomponent drugs. Even if both strategies are effective, polypharmacology possesses important features to underline. Firstly, the use of only one molecule instead of many is conceivably associated with a possible lower cytotoxicity and side effects. Moreover, polypharmacology is expected to have a higher therapeutic efficacy, compared to the classical approach of hitting only one best target at a time [13]. Moreover, multitarget drugs show synergic or additive effects, this means that they can modulate complex diseases in lower time and with smaller doses thanks to simultaneous targeting [14]. Lastly, they may avoid the problem of drug–drug interaction. All these features allow the consequential improvement of the patient quality of life [15].

**Figure 1:**
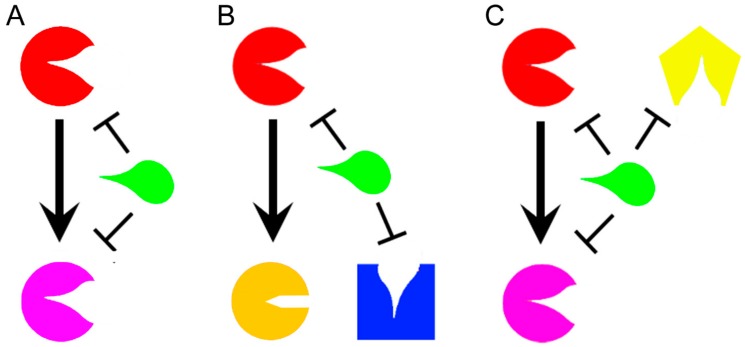
Representation of a general drug (green) that acts in vertical targeting in series (**A**) when it inhibits two related enzymes or in parallel (**B**) when it targets two unrelated enzymes with a common substrate. A drug acts in network inhibition (**C**) when it targets different enzymes in different metabolic pathways. The different targeted enzymes are arbitrary represented in different colors (Red, Pink, Yellow, Orange, and Blue).

For these reasons, polypharmacology is an emerging strategy in therapeutic development of drugs against synergistic bacterial diseases [16], neurological diseases [17], and cancer [18]. Even if the development of multitarget drugs is a relatively novel field, some classifications have been already proposed according to their mechanisms of action, or their structures. Based on mechanisms of action, a molecule that affects different targets within the same metabolic pathway acts in “vertical targeting”. We can distinguish a “series inhibition” if the two targets are related (Figure 1A), as for example consequential or in the same pathway, while a “parallel inhibition” (Figure 1B) when they are unrelated, but should have for example a common substrate that can be mimicked [12]. The vertical targeting is a strategy that can fight the insurgence of certain kinds of resistance mechanisms such as mutations [19]. A drug that acts in “network targeting” instead, hits different targets in different pathways (Figure 1C) [12], and it is able to prevent compensatory homeostatic responses and the adaptive resistance [14]. 

**Figure 2:**
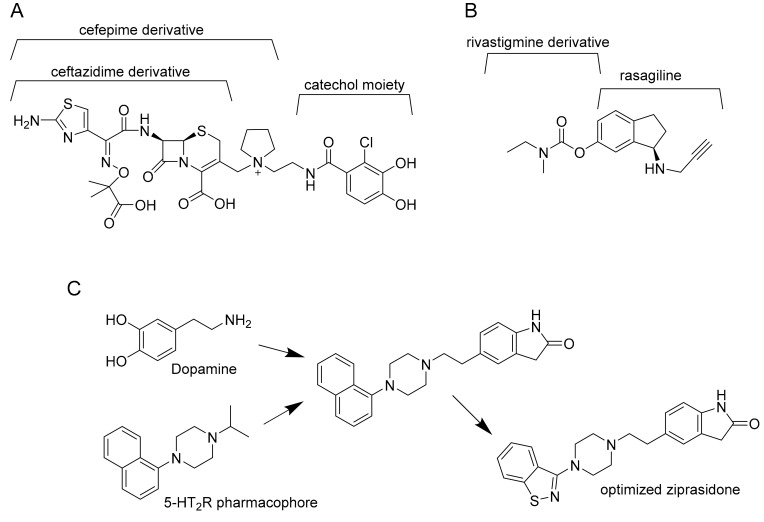
Example of linked (**A**), fused (**B**), and merged pharmacophores (**C**). Cefepime derivative is the linker between ceftazidime derivative and catechol moiety in cefiderocol (**A**). Ladostigil, an analogue of compound 9, is derived from the fusion of rasagiline with rivastigmine (**B**). Finally, dopamine is merged with 5-HT_2_R pharmacophore and the obtained molecule is optimized to ziprasidone (**C**).

By structure, multitarget drugs may be classified according to the optimization and modifications that are introduced by chemical tailoring to the original molecule. Another possibility is to rationally design a multitarget drug by virtual studies. This strategy gives rise to three possibilities: Linked, fused, or merged pharmacophores (Figure 2).

Linked pharmacophores are molecules bound together by a stable or biodegradable linker [20]. The result is a larger molecule that does not need further improvement, but the position of the linker is crucial for its final effect on target. Moreover, this molecule may fail to reach the intracellular compartment of interest because of its size. From another point of view, some linkers can improve solubility and polarity of the molecule and reduce unspecific diffusion into an unwanted cellular compartment [21]. Nonetheless, linked pharmacophores are currently used to produce antibody drug conjugates, that is, a drug (usually a small molecule) conjugated with an antibody that works as a vessel to reach the target. Another example is to conjugate a drug, as for example an antibiotic, to bacterial siderophores. The resulting sideromycin exploits the bacterial uptake mechanisms to be internalized. A promising example is Cefiderocol. This compound, that binds the penicillin-binding protein 3 (PBP3) inhibiting cell wall biosynthesis, is active against different multidrug-resistant Gram-negative pathogens such as carbapenem-resistant *Pseudomonas aeruginosa, Acinetobacter baumannii*, and *Enterobacteriaceae,* and is currently in phase II clinical trials [22]. 

Fused pharmacophores are the result of two joint small molecules without a linker. According to the type of final bond (imine, ester, or hydrazine bonds) they may be cleavable or not. Moreover, the position of the bond is crucial for the final effect of both molecules and the resulting pharmacophore may be a large molecule with all the consequent problems for its delivery. According to Sterling et al. [23], for example, the “compound 9” described in their article is active against both acetylcholinesterase (AChE) and monoamine oxidases (MAOs), and it was created by fusion of rivastigmine and rasagiline [23]. 

Finally, merged pharmacophores are possible only when a part of two different molecules can overlap, resulting in their integration. Anyway, to be active, the resulting molecule must possess the correct molecular geometry and charge distribution to interact with both targets [24]. For example, Ziprasidone is an antipsychotic drug optimized by the resulting fusion of dopamine and 5-HT_2_R pharmacophore. It targets both type 2 dopamine receptors D2 and type 2 serotonin synergic receptors 5-HT_2_R [25].

## 3. Multitargeting Compounds Against *M. tuberculosis*

The single-target strategy for the development of novel antimicrobial drugs involves single proteins essential for survival of the pathogen. However, this approach shows a weakness, since a single mutation in the target protein could be sufficient to confer resistance. For this reason, drug combinations in TB treatment are preferred over single-drug therapies; in this context multitarget drug discovery may offer a novel opportunity [12]. In recent years, several multitargeting antitubercular compounds have emerged [26]. Many compounds have been firstly discovered through phenotypic screening, then they were retrospectively found to target multiple proteins simultaneously, such as the case of the MmpL3 inhibitors [12]. Interestingly, in some cases the compound has been designed or selected as an inhibitor of a specific enzyme or pathway, then it was found to have additional targets. For instance, the recently described 5-(5-nitrothiophen-2-yl)-4,5-dihydro-1*H*-pyrazoles, have been selected as potential inhibitors of the arylamine *N*-acetyltransferase enzyme, and were found to be also potent efflux pump inhibitors [27,28]. Another example is the case of the tetrahydroisoquinoline compounds, that have been selected as inhibitors of the ATP-dependent MurE ligase, but were found to have pleiotropic mechanisms of action, not fully clarified yet [29]. On the other hand, compounds are also emerging from biochemical screens against specific targets, as for instance the GroEL/ES chaperonin and protein tyrosine phosphatase B inhibitors [30]. Finally, multitargeting compounds have been specifically designed, as for instance the fatty acids bypass biosynthetic pathways inhibitors [31]. The most recent and significant examples of the different multitargeting antitubercular compounds are here described. 

### 3.1. MmpL3 Inhibitors 

**Figure 3:**
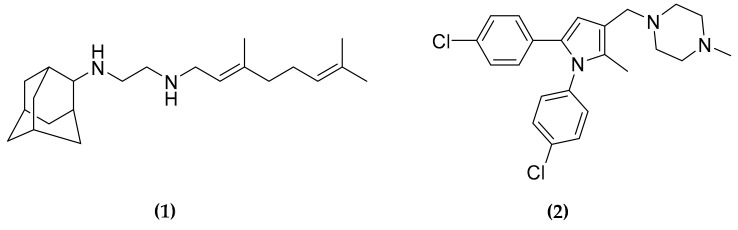
Structure of trehalose monomycolate transporter (MmpL3) inhibitors SQ109 and BM212.

The trehalose monomycolate (TMM) transporter MmpL3 is an essential protein involved in the translocation of TMM and cell wall mycolates across the membrane. This protein has been defined as a promiscuous target since several compounds with different scaffolds have been demonstrated to affect its activity [32]. Among the reported MmpL3 inhibitors, a very promising compound is SQ109 **(1)** (Figure 3), a derivative of ethambutol but with a different mechanism of action with respect to its original structure [33]. Actually **(1)** is now in phase II clinical trial [34]. 

As the substrates of MmpL3 are part of the cell wall structure, **(1)** seems to act as a cell wall inhibitor [35]. However, the direct inhibition of MmpL3 has been debated, since **(1)**, as well as another inhibitor, the 1,5-diarylpyrrole BM212 **(2)** (Figure 3), have been shown to be active also against latent MTB and against other bacterial species lacking this transporter [36]. For this reason, an indirect effect on MmpL3 translocation was suggested, due to the disruption of the proton motive force (PMF) driven by these compounds. Moreover, Li et al. [36] demonstrated that **(1)** and some derivatives inhibit in vitro two other MTB enzymes, MenA and MenG, both involved in the biosynthetic pathway of quinone structures. MenA catalyzes the formation of demethylmenaquinol by isoprenylation of 1,4-dihydroxy-2-naphthoic acid using one molecule of isoprenoid diphosphate. MenG acts directly on demethylmenaquinol, catalyzing its S-adenosylmethionine-dependent methylation to synthesize the demethylmenaquinone. Since this quinone is involved in electron transport, the final effect is an impairment of ATP biosynthesis [36].

However, the binding of several inhibitors to MmpL3 has been recently demonstrated, using a fluorescent competition assay and surface plasmon resonance with the purified protein [37], further confirmed by the crystal structure of the protein in complex with different compounds, **(1)** included [38]. Nevertheless, among these inhibitors only **(1)** and **(2)** were demonstrated to dissipate both ΔpH and ΔΨ, thus confirming the effects of these two compounds on the PMF [38]. 

It is worth noting that all the attempts to directly isolate any spontaneous resistant MTB mutant to these two compounds failed [39,40], further demonstrating the usefulness of compounds that have multiple targets in preventing the resistance insurgence.

### 3.2. Designed Dual Inhibitors Against Fatty Acids Bypass Biosynthetic Pathways

**Figure 4:**
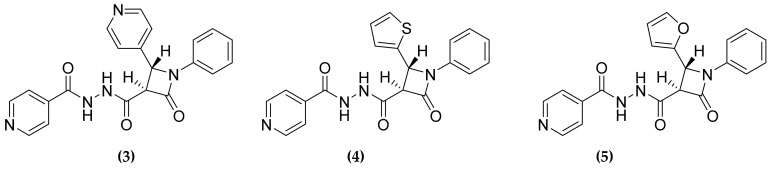
FabG4 and HtdX dual inhibitors.

*Mycobacterium tuberculosis* possesses a very thick cell wall that allows a certain resilience to molecule diffusion inside the cell and is essential for the survival during the host infection. One main component of the cell wall is a plethora of fatty acids that are synthetized primarily by the fatty acid synthesis (FAS) pathway. MTB possesses two main pathways: FAS-I, a multidomain enzyme that catalyzes the synthesis of short-chain fatty acids, and the FAS-II system involved in long-chain fatty acids production [41]. Some fatty acids, anyway, depend on alternative bypass biosynthetic pathways, such as for example the metabolism of interlinked CoA dependent fatty acid [42], which could represent an attractive target for novel antitubercular compounds. In this context, Banerjee et al. selected the two enzymes, encoded on a single operon, FabG4 and HtdX for the development of novel multitargeting compounds [31]. The first enzyme is a β-ketoacyl CoA reductase, while the second is a 3(R)-hydroxyacyl CoA dehydratase that acts on the reduced ketoacyl, the product of the reaction catalyzed by FabG4. Both enzymes are reported as essential for MTB survival [41,42,43] and being involved in consecutive steps of a metabolic pathway, a common inhibitor is supposed to have synergistic effects. To this purpose, the authors used a blended structure-based and ligand-based design approach. Firstly, based on the structural information of the catalytic sites they selected as pharmacophores the β-lactam and the isoniazid scaffolds, as well as several aromatic rings. The pharmacophores were then combined to form a small library, that was used for docking studies to select the potential inhibitors. Through this analysis they selected seven scaffolds, three of them showing significant activity against both enzymes (Figure 4). 

Moreover, the three compounds displayed interesting antimycobacterial activity against *M. smegmatis*, both planktonic and in biofilm, demonstrating the validity of this approach to achieve novel drug candidates [28].

### 3.3. Oxadiazolone Derivatives Targeting (Ser/Cys)-Enzymes

**Figure 5:**
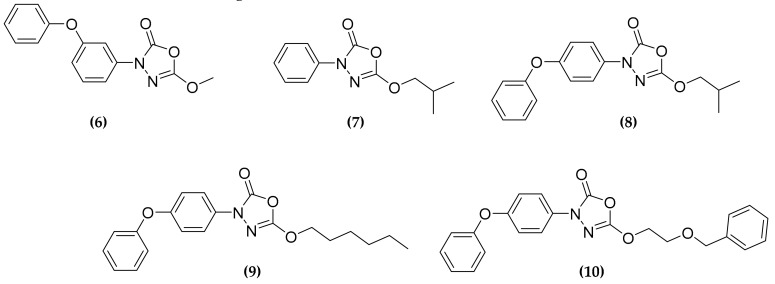
Oxadiazolones inhibitors of Ser/Cys-based enzymes, showing both in vitro and ex vivo antitubercular activity.

The oxadiazolone core is an interesting scaffold, which characterizes compounds known to have antimycobacterial activity [44,45]. Among these compounds, 5-methoxy-3-(3-phenoxyphenyl)-1,3,4-oxadiazol-2(3*H*)-one **(6)** (Figure 4), was demonstrated to be an inhibitor of the hormone-sensitive lipase family of enzymes, which forms a covalent slowly reversible bond, such as carbamate or thiocarbamate, with the catalytic serine or cysteine residue [46]. Thus, since lipolytic enzymes are important for pathogen and host cross-talk, for pathogenic reactivation or survival during dormancy state, it could represent an interesting scaffold for the development of therapeutic compounds [47]. Recently, starting from these results, Nguyen et al. developed eighteen oxadiazole derivatives, by modifying the R chain and the positioning of the phenoxy group [48]. Four compounds **(7**–**10)** (Figure 5) displayed moderate activity against MTB growth in vitro, but demonstrated to be very active ex vivo, in a macrophage infection model, with low µM minimal inhibitory concentration (MIC) values, although not toxic for the host. 

In order to identify the target(s) of the compounds, the researchers applied an activity-based protein profiling (ABPP) approach, that allowed the identification of 18 different proteins that bind oxadiazolone derivatives. As expected, all the identified proteins were Ser/Cys-based enzymes, including several hydrolases, five of them reported as essentials [48]. Three of these putative targets, the proteins TesA, Cfp21, and Rv0183, have been expressed in recombinant forms, and were confirmed to be directly inhibited by the compounds. This work allowed the identifications of novel unexploited pathways and enzymes as potential targets for novel antitubercular multitargeting compounds [48].

### 3.4. CTP and CoA Biosynthesis Inhibition

**Figure 6:**
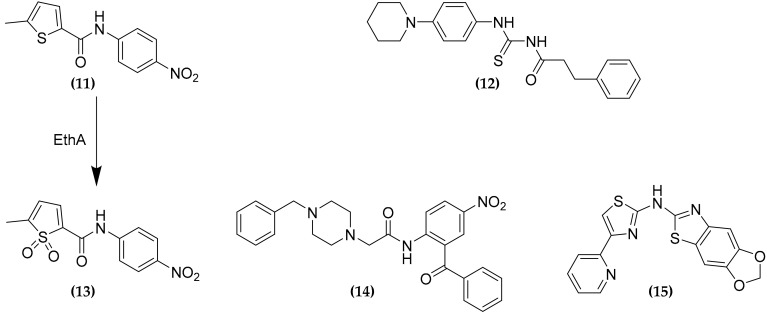
Dual PyrG-PanK inhibitors.

One advantage of multitargeting compounds is that the simultaneous inhibition of two different interconnected pathways should lead to synergistic effects. This is the case for the PyrG and PanK inhibitors. The two compounds 5-methyl-*N*-(4-nitrophenyl) thiophene-2-carboxamide **(11)** and 3-phenyl-*N*-[(4-piperidin-1-ylphenyl)carbamothioyl]propanamide **(12)**, have been firstly identified in a high throughput phenotypic screening study as inhibitors of the CTP synthetase PyrG [49]. Both compounds were demonstrated to be indeed prodrugs, which need activation by the monooxygenase EthA to exert their antimycobacterial activity, although only for **(11)** the active sulfone product **(13)** has been identified (Figure 6). Moreover, during further investigations, these compounds were found to possess at least another target, being able to inhibit the pantothenate kinase PanK [50]. This enzyme catalyzes the first step of coenzyme A (CoA) biosynthesis, by conversion of the pantothenate (vitamin B5) into 4′-phosphopantothenate. Moreover, two other compounds **(14)** and **(15)**, identified through in silico and in vitro target based screenings against PyrG inhibitors, respectively, were demonstrated to inhibit also the PanK activity [49,50,51]. CoA is an essential cofactor for many key enzymes of different metabolic pathways, such as fatty acids biosynthesis and catabolism, for this reason limiting its bioavailability results in an attractive therapeutic strategy. 

Nevertheless, target knockdown studies demonstrated that PanK is not useful as a potential drug target, showing poor vulnerability [52]. However, it is worth noting that, although **(15)** is a relatively moderate inhibitor of the two enzymes, with IC_50_ values about 5-fold higher than **(13)**, the compound has a similar MIC value against MTB. Moreover, metabolic labelling studies demonstrated a similar impairment in lipid metabolism, which derives from the combined depletion of activated CDP-derivatives, necessary for the biosynthesis of phospholipids, and decreased CoA levels [50]. This work thus confirmed how polypharmacology should improve the efficacy of compounds, through the synergistic effect of the simultaneous inhibition of two targets.

### 3.5. Salicylanilide Carbamate Compounds 

Salicylanilide derivative compounds have multiple biological targets described in literature such as d-Ala-d-Ala ligase [53], transglycosidase [54,55], isocitrate lyase and methionine aminopeptidase [56], L-alanine dehydrogenase, lysine -aminotransferase, chorismate mutase, pantothenate synthetase [57], and they are also able to act as uncouplers disrupting the proton gradient [58]. Moreover, many compounds were found to be active against MTB, as well as against different nontuberculous mycobacterial strains, with MIC values in order of few micromolar or submicromolar [59].

**Figure 7:**
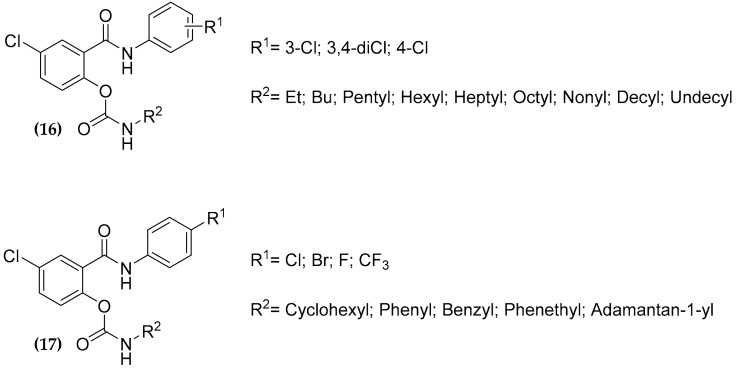
Antitubercular salicylanilide carbamide compounds.

Since carbamates have been reported useful to protect phenolic drugs [60], Férriz et al. masked the phenolic hydroxyl salicylanilide through carbamate formation, in order to increase the hydrophobicity of the structure thus putatively conferring a better permeability through the MTB cell wall, achieving a series of salicylanilide *N*-*n*-alkyl carbamate compounds **(16)** (Figure 6) with MIC values ranging from 0.5–2 µM [61]. Subsequently, further *N*-cycloalkyl/phenyl/phenylalkyl carbamates have been synthetized [62,63,64]. This series (compounds **(17),** Figure 7) showed significant antibacterial and antitubercular activity [59,63], with a reduced cytotoxicity, although the selectivity in many cases remained rather low [63]. 

Several salicylanilide derivatives are known to target, among others, the isocitrate lyase ICL1, an enzyme involved in the glyoxylate and methylcitrate cycles, important for MTB persistence, thus offering an advantage in fighting TB at different stages [65]. For this reason, the compounds belonging to the **(17)** series have been assayed against the recombinant enzymes but resulted only in a very weak inhibition of the activity [59]. Thus, the actual targets of these very promising antitubercular compounds are still to be clearly identified.

### 3.6. Dualtargeting GroEL/ES Chaperonin and Protein Tyrosine Phosphatase B

**Figure 8:**
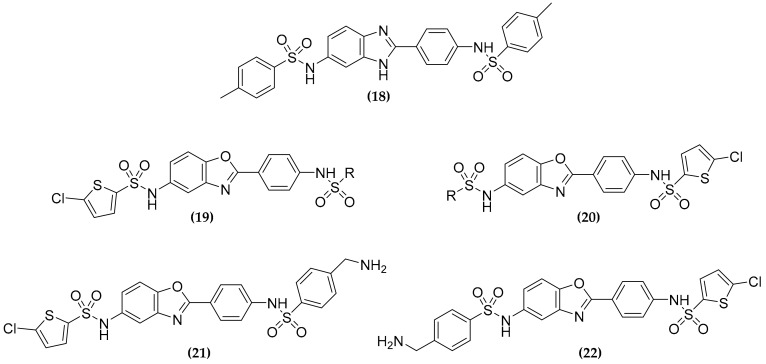
Structure of the GroEL/ES chaperonins (GroEL/GroES) and protein tyrosine phosphatase B (PtpB) dual inhibitors.

Protein homeostasis pathways, particularly molecular chaperones are emerging targets for antimicrobial drug discovery [66]. *M. tuberculosis* possesses two different GroEL/ES chaperonins (GroEL1 and GroEL2) that, together with proteases, maintain cellular protein homeostasis, the former helping proteins to fold, while the latter committed to their proper degradation. Among the two chaperonins, that have a sequence identity of only 61%, only GroEL2 is essential for MTB survival, while GroEL1 is important for the granuloma formation. Thus, the simultaneous inhibition of these two proteins should be efficacious against both active and latent TB [67]. To this purpose, Johnson et al. in 2014 performed a biochemical screen of 700,000 small molecules, identifying 235 compounds that inhibit GroEL/GroES-mediated refolding [68], 22 of them have been then evaluated for the antibacterial activity [69]. Starting from the benzimidazole based compound **(18)** (Figure 8), the authors developed a series of derived benzoxazoles **(19)**, with variable sulfonamide end caps [70], showing good activity against Gram positive bacteria [71]. Based on these results, these compounds have been evaluated against MTB, but, to determine if simplifying these inhibitors could reduce their cytotoxicity, the “half-molecules” containing only one sulfonamide end-capping on either right or left sides of the molecule, have also been developed [30]. However, the “full-molecules” were always more potent than the “half-molecules”, confirming the importance of both aryl-sulfonamide moieties for inhibition.

Furthermore, the authors noticed some similarity within these compounds and the manner in which the two (oxalylamino-methylene)-thiophene sulfonamide (OMTS) molecules bind the active site of their target, the protein tyrosine phosphatase B (PtpB) [30]. Indeed, the analysis of the crystal structure of PtpB in complex with the inhibitor, revealed that the sulfonamides moiety of each OMTS molecule were ∼ 11–12 Å apart, similarly to the **(19)** compounds, suggesting that they could bind the protein, bridging the distal and proximal parts of the active site [30]. PtpB is an MTB phospho-tyrosine phosphatase secreted into the macrophages cytoplasm that interacts and blocks Erk1/2, p38 mediated IL-6 production, and the Akt signaling, subsequently causing the interference of macrophages immune response, and promoting intracellular survival [72]. In light of this evidence a dual targeting compound able to inhibit both GroEL/ES chaperonin systems and PtpB, should be an effective strategy to treat all stages of tuberculosis. With this aim, two compounds were identified **(21)** and **(22)**, characterized by a 5-chlorothiophene and a primary amine either on the right or left-hand sides of the structure [30]. 

These compounds showed moderate antitubercular activity, but a good selectivity over the human counterpart of the target enzymes, being a promising starting point for the development of more potent multitargeting inhibitors [30].

### 3.7. Multitargeting of the Folate Pathway

Folate metabolism has been recently considered an interesting target for potential antitubercular compounds, as this cofactor is essential for several pathways, such as the synthesis of methionine, purines, and of the deoxythymidine monophosphate. 

**Figure 9:**
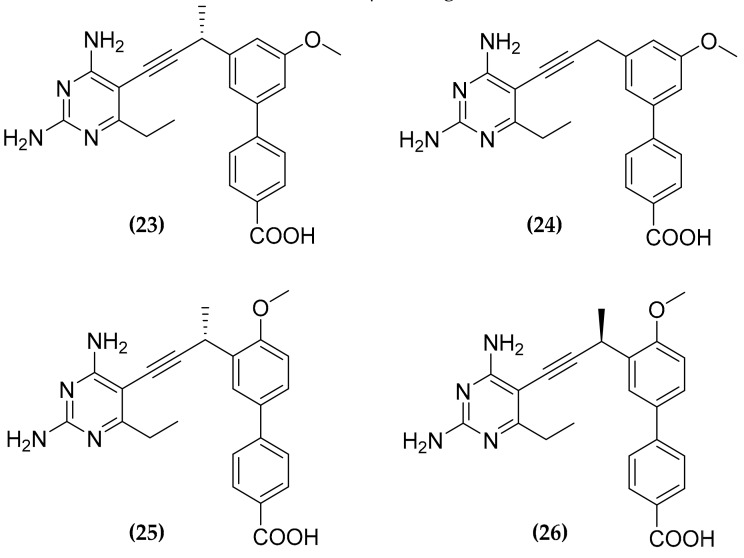
Structure of the antitubercular folate pathway inhibitors ionized nonclassical antifolates (INCAs).

Folate antimetabolites, or antifolates, should block the production of reduced folate through the inhibition of the key enzymes in these metabolic pathways. Among these enzymes, dihydrofolate reductase (DHFR), that catalyzes the reduction of dihydrofolate to tetrahydrofolate has gained interest, as a target for anticancer and antimicrobial therapies [73]. Nevertheless, the currently used antifolates, are not particularly active against MTB, for several reasons. For instance, trimethoprim has a low affinity for the mycobacterial DHFR, while other compounds, such as methotrexate or pyrimethamine show good potency against the target in vitro but are characterized by a low cell membrane permeability [74]. However, promising antifolates are the propargyl-linked antifolates (PLAs) designed as inhibitor of the Staphylococcus Aureus DHFR [75]. As these compounds are supposed to enter cells through passive diffusion, they were supposed to have good permeability also over the MTB cell wall. Indeed, a screening of a PLAs library identified a series of active compounds, that were named ionized nonclassical antifolates (INCAs) **(23)**–**(26)** (Figure 9) [76]. 

Interestingly, these compounds have been shown to inhibit also an alternate folate pathway [77], which relies on two recently discovered enzymes: Rv2671 the second DHFR found in MTB, and the flavin-dependent thymidylate synthase (FDTS) [78,79]. In particular, INCAs showed good activity against both DHFR and Rv2671, with an inhibitory constant in the low nM range, and good selectivity over the human dihydrofolate reductase. Thus, inhibiting two enzymes belonging to an alternate pathway for the same metabolite, INCAs frustrate the functional redundancy that should ensure high levels of reduced folates, leading to considerable potency against MTB, with MIC values in the nM-low µM range [76].

Moreover, a special mention is mandatory for the *para*-aminosalicylic acid (PAS). Indeed, PAS targets DHFR as a prodrug, and its metabolite PAS-M has shown to act as a competitive substrate for two other enzymes: The dihydropteroate synthase DHPS along the same biosynthetic route and the flavin-dependent thymidylate synthase FDTS in the alternative one [77]. Nowadays, PAS is the only successfully used inhibitor of folate pathway in TB treatments.

### 3.8. Ethionamide and Ethionamide Booster Co-Administration

Several antitubercular compounds, including drugs in clinical practice, are indeed prodrugs, that need enzymatic activation to fulfill their inhibitory effects [80]. Ethionamide (ETH) **(27)**, one of the most widely used second-line drugs for MDR-TB treatment, is converted into the nicotinamide adenine dinucleotide adduct by the Bayer–Villiger monooxygenase EthA. Then, the active adduct acts inhibiting the enoyl-acyl-carrier-protein reductase InhA, impairing the mycolic acids biosynthesis [81]. Since EthA is regulated by the transcriptional repressor EthR, the limited expression of the activator can limit the bioactivation of **(27)**. Thus, to overcome this issue several EthR inhibitors have been developed, that allowed to greatly increase the in vivo potency of **(27)** [82,83,84]. 

**Figure 10:**
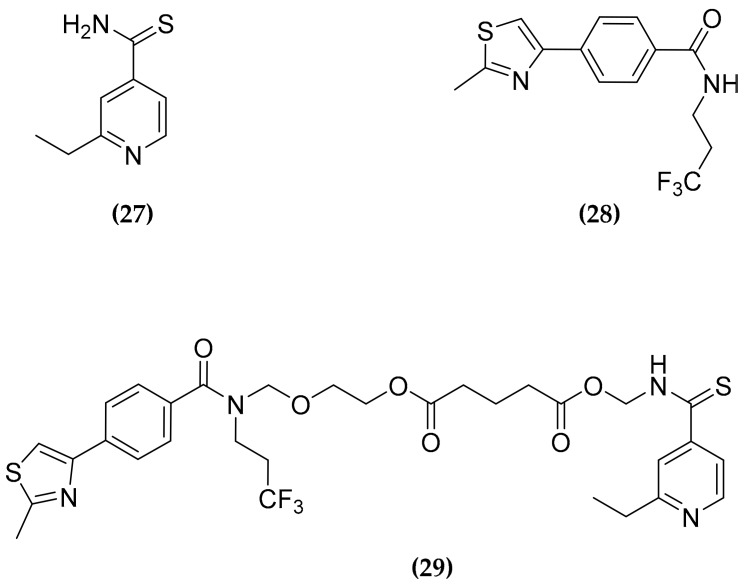
Structure of the second-line antitubercular drug ethionamide (ETH) **(27),** of its booster **(28)**, and of the co-drug **(29)**.

However, the co-administration of ETH and booster have been found to be hampered by several factors, particularly the low water solubility of these compounds and the propensity of **(27)** to crystallize. To address the issue of the solubility of the compounds, β-cyclodextrin based nanoparticles for the simultaneous co-administration of **(27)** and the BDM43266 **(28)** booster (Figure 10) have been developed [85]. However, these nanoparticles displayed drug loading only of 5%. A great improvement was achieved by the synthesis of a co-drug **(29),** able to self-associate into nanoparticles (Figure 10) [86]. 

The new compound was obtained by tethering N-hydroxymethyl derivatives of both **(27)** and **(28)** through a glutaric linker, potentially cleavable intracellularly by esterase. Interestingly, **(29)** was found able to self-assemble into nanoparticles in a very stable way, which displayed a drug loading of about 80%. Moreover, a suspension of these nanoparticles was found very active in a mouse model, upon direct intranasal administration into the lung [86]. This compound represents a further example of the flexibility and effectiveness of the multitargeting approach.

### 3.9. In Silico Approaches for Multitargeting Compounds Development

The development of effective multitargeting compounds could benefit also from in silico approaches. For instance, Janardhan et al. proposed and implemented an in silico guided polypharmacological approach, based on a combination of pharmacophore and QSAR based virtual screening strategy [87]. Starting from three well recognized antitubercular drug targets, such as InhA, the *N*-acetyl-glucosamine-1-phosphate uridyltransferase GlmU, and the dihydrodipicolinate reductase DapB, they selected 784 hits from a large database, by structure based and ligand based virtual screening protocols. These structures were then further subjected to docking studies against 33 potential targets, and the 110 potential multitargeting hits identified were subjected to different screening protocols to evaluate different parameters, including cell permeability, drug-likeness, and structural alerts. Finally, they achieved nine structures potentially active against more than 10 different targets, as scaffolds for future designs of selective inhibitors, although to date none of them have been assayed yet [87]. However, considering this approach, Volynets et al. [88] identified a hit compound that efficiently inhibits in vitro the two enzymes leucyl-tRNA synthetase LeuRS and methionyl-tRNA synthetase MetRS, thus confirming the usefulness of in silico studies to develop multitargeting antitubercular agents [88].

## 4. Conclusions and Future Perspectives

The global problem of the spread of MDR and XDR MTB strains leads to the necessity of novel approaches for the development of the so called “resistance resistant” drugs. In this context polypharmacology appears very promising, as demonstrated by the numbers of compounds that have been designed or discovered in recent years, here described. Moreover, it is worth noting that the two compounds PAS and SQ109, that are already in clinical practice or trials for TB treatment, have been demonstrated to be multitargeting compounds, thus demonstrating the great potential of this approach for the development of new drugs to eradicate TB, and contrast the spreading of resistant MTB strains.
